# Congenital sinus node dysfunction and atrial fibrillation in pediatric short QT syndrome type 2: Implications for pacing strategy

**DOI:** 10.1016/j.hrcr.2026.07.019

**Published:** 2026-07-31

**Authors:** Maria Angela D. Magpantay, Seshadri Balaji, Anthony C. McCanta, Lauren Sell, Joseph Spindler, Anjan S. Batra

**Affiliations:** 1 University of California Irvine School of Medicine, Irvine, California; 2 Department of Pediatric Cardiology, Oregon Health and Science University, Portland, Oregon; 3 Department of Pediatric Cardiology, University of California Irvine School of Medicine, Irvine, California; 4 Cardiology, Rady Children’s Health, Orange, California

**Keywords:** *KCNQ1* p.Val141Met, Short QT syndrome, Pediatrics, Sinus node dysfunction, Atrial fibrillation, Bradycardia, Pacemaker, Genetics

**Key Teaching Points**

• Short QT syndrome type 2 (SQT2) is associated with perinatal bradycardia, sinus node dysfunction, and early-onset atrial fibrillation (AF).
• Fine AF in infants can mimic electrical artifact on surface electrocardiogram, making recognition challenging.
• Persistent AF can render atrial pacing ineffective despite normal lead parameters and should be considered when atrial lead noncapture occurs. Ventricular pacing may ultimately be required in selected patients with SQT2 and persistent AF when atrial pacing becomes ineffective.

## Introduction

Short QT syndrome (SQTS) is a rare, inherited arrhythmia characterized by shortened QT intervals on electrocardiogram (ECG) and an increased risk of both atrial and ventricular arrhythmias.^1^ The 2022 European Society of Cardiology diagnostic criteria include a corrected QT interval (QTc) of ≤360 ms in the presence of additional features, such as a pathogenic mutation, family history of SQTS, or documented survival from a ventricular tachycardia/ventricular fibrillation in the absence of structural heart disease.^2^ Another form of diagnostic criterion is the modified Gollob score, which is a point-based scoring system assigned to the ECG findings (QTc <370 ms and the J point–to–T peak interval), family and medical history, and genetic findings.^3^ SQTS is strongly linked with multiple pathogenic mutations affecting cardiac ion channels, which are located in 4 main genes: 3 encoding potassium ion channels—*KCNH2*, *KCNJ2*, and *KCNQ1*—and 1 encoding a chloride–bicarbonate anion exchanger, *SLC4A3.*^4^^,^^5^ It has been previously reported that such variants exhibit autosomal dominant inheritance. However, de novo mutations do exist.^4^^,^^6^

SQTS type 2 (SQT2) results from gain-of-function mutations in *KCNQ1*, which encodes the α-subunit of the slow delayed rectifier potassium current channel.^1^ Enhanced slow delayed rectifier potassium current shortens cardiac action potential duration and facilitates both atrial and ventricular arrhythmogenesis.^1^ Within the *KCNQ1* gene, 2 pathogenic variants have been identified to produce SQTS with the concomitant atrial fibrillation (AF) phenotype: p.Val141Met and p.Val307Leu, with the former being associated with significantly slower sinoatrial pacemaker activity.^4^^,^^7^ Fewer than 20 cases of SQT2 have been reported.^6^ In pediatric patients, sinus node dysfunction is a common early manifestation.^6^^,^^8^ SQT2 has also been associated with fetal bradycardia and early-onset AF.^9^

Because of the rarity of this condition, optimal management strategies remain poorly defined. We describe 2 unrelated pediatric patients with genetically confirmed SQT2 who presented with congenital sinus node dysfunction and subsequently developed high-frequency AF, highlighting diagnostic challenges and important pacing considerations.

## Case report

### Case 1

A male infant was delivered at 34 weeks’ gestation via emergent cesarean section for fetal bradycardia and suspected placental abruption. After delivery, he developed acute pulmonary hypertension and persistent bradycardia with heart rates in the 50–60 beats per minute (bpm) range. ECG demonstrated severe bradycardia with junctional rhythm, absent discernible P waves, and a shortened QT/QTc of 336/307 ms using Bazett correction (heart rate approximately 50 bpm), which persisted on serial ECGs (Figure 1). Fine, high-frequency atrial activity was initially interpreted as artifact. Heart rate improved marginally to the 70s with isoproterenol and dobutamine therapy.

**Figure 1:**
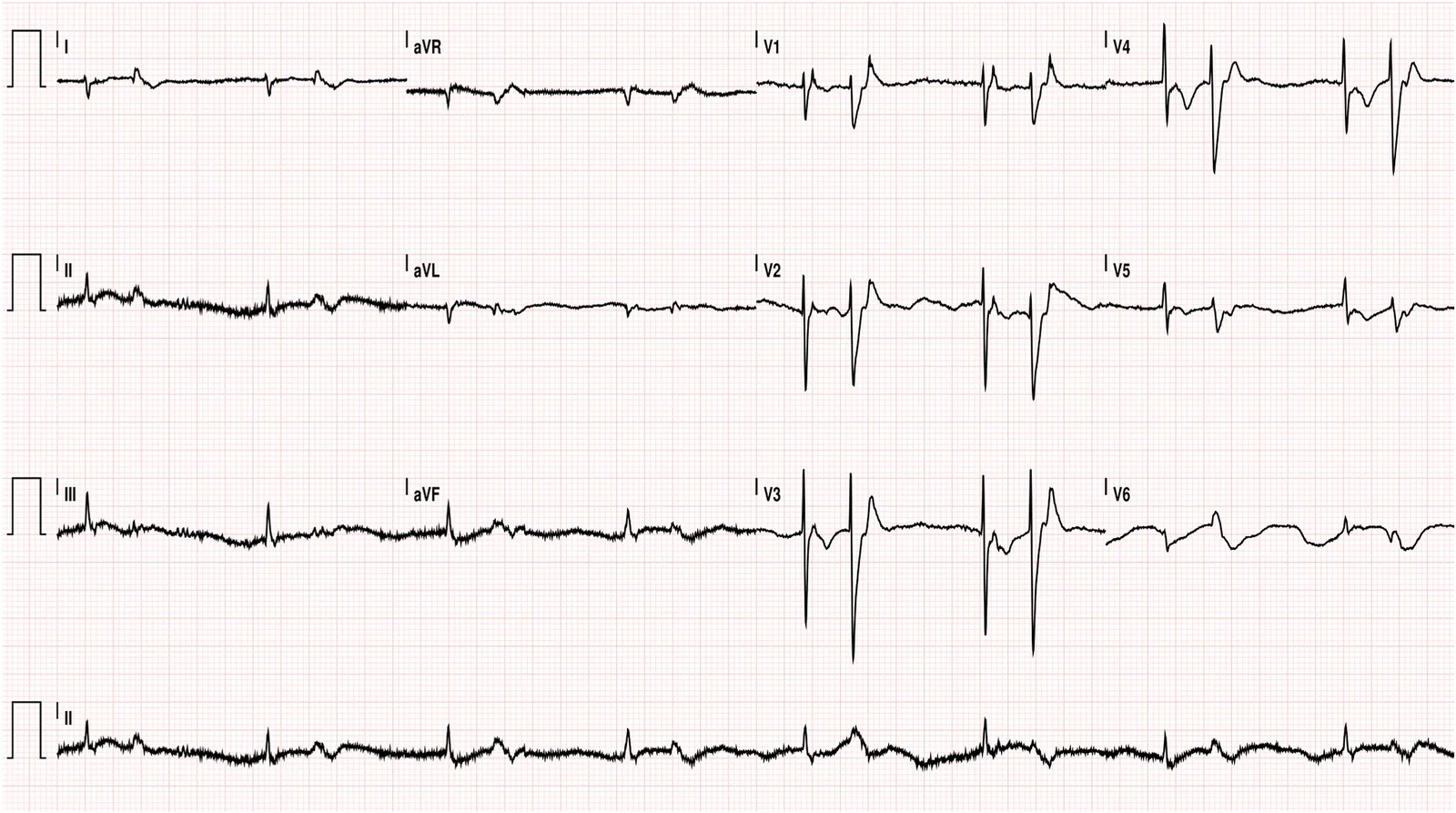
Electrocardiogram at 5 hours of life (case 1). Demonstration of sinus bradycardia, a corrected QT interval of 307 ms, and high-frequency atrial activity interpreted as artifact.

The patient underwent transesophageal pacing for 3 days, followed by implantation of an atrial pacemaker programmed AAIR. Chromosomal microarray testing was normal. Whole-genome sequencing subsequently identified a heterozygous *KCNQ1* p.Val141Met mutation, consistent with SQT2. Maternal genetic testing was negative for the variant. Paternal testing was unavailable because consent was declined. Implantable cardioverter-defibrillator implantation was discussed but deferred given the absence of ventricular arrhythmias, syncope, or family history of sudden cardiac death.

At 6 months of age, atrial lead noncapture was observed at maximum output despite stable lead impedance. High-frequency atrial signals were interpreted as lead noise. Lead fracture was initially suspected. Given the patient’s clinical stability and the presence of an adequate junctional escape rhythm, device revision was deferred. At 19 months of age, ambulatory monitoring demonstrated persistent junctional rhythm with average heart rates of 40–50 bpm, along with high-frequency atrial activity (Figure 2). The patient was referred for implantation of a dual-chamber epicardial pacing system.

**Figure 2:**
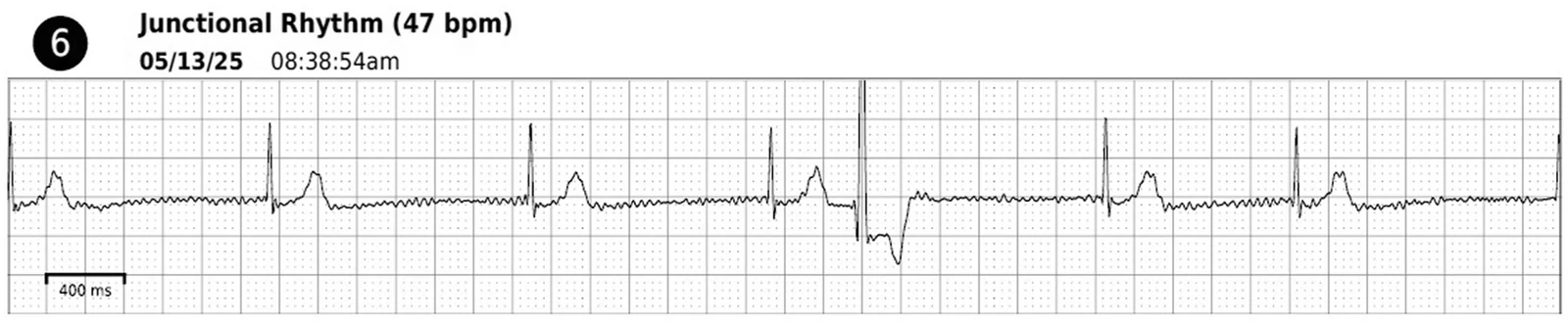
Ambulatory monitor at 19 months of age (case 1). Junctional rhythm and high-frequency atrial activity are noted. bpm = beats per minute.

Intraoperative attempts at atrial lead placement were unsuccessful despite electrical cardioversion, raising concern for persistent AF or atrial standstill. A dual-chamber epicardial system was therefore implanted and programmed VVIR. Postoperative device interrogation demonstrated rapid, irregular atrial electrical activity consistent with high-frequency AF (Figure 3). The postoperative course was uncomplicated, and the patient was discharged on aspirin therapy. Aspirin therapy was prescribed after multidisciplinary discussion because the thromboembolic risk associated with persistent AF in young children with SQT2 is unknown and pediatric anticoagulation strategies are not well defined. However, we acknowledge that evidence-based antithrombotic management in these patients remains to be investigated. He remains clinically stable at 2 years of age.

**Figure 3:**
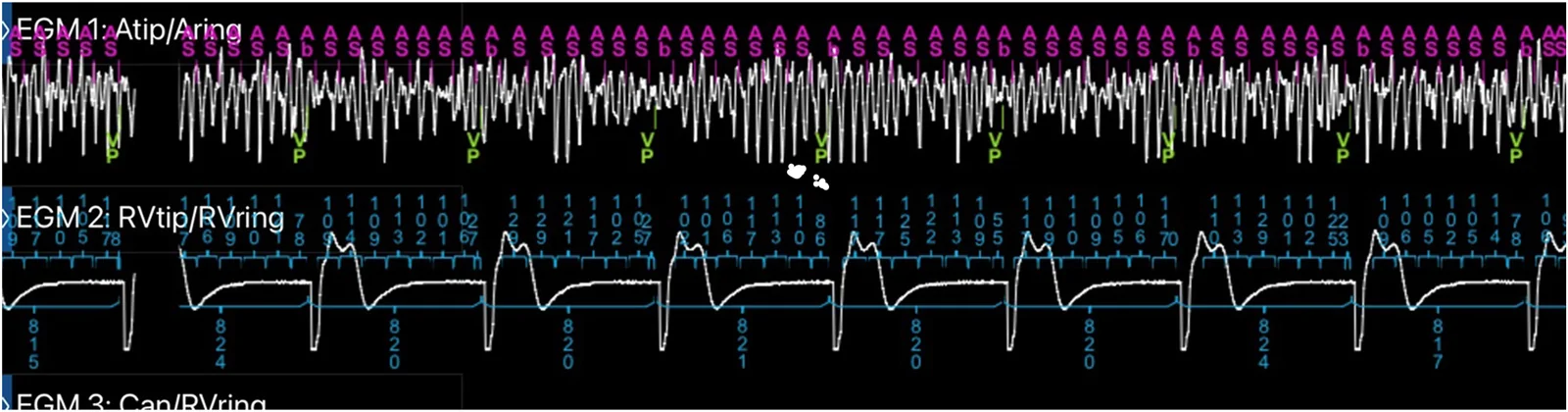
Device interrogation on VVIR pacing at 2 years of age (case 1). Underlying high-frequency atrial activity on atrial channel in pacemaker (A-A interval) consistent with persistent atrial fibrillation. EGM = electrogram.

### Case 2

A male infant was delivered at 37 weeks’ gestation via scheduled cesarean section for fetal bradycardia first identified at 26 weeks’ gestation. Neonatal ECG demonstrated severe bradycardia with a junctional rhythm consistent with sinus node dysfunction and a shortened QT/QTc of 260/314 ms using Bazett correction (heart rate approximately 60 bpm) (Figure 4). Serial ECGs demonstrated persistently shortened QT intervals. The patient remained hemodynamically stable without the need for respiratory or pharmacologic support. Echocardiography revealed a small patent ductus arteriosus, biventricular dilation, and preserved left ventricular function. An atrial pacemaker was implanted using a unipolar atrial lead, and the device was programmed to AAIR owing to preserved atrioventricular conduction.

**Figure 4:**
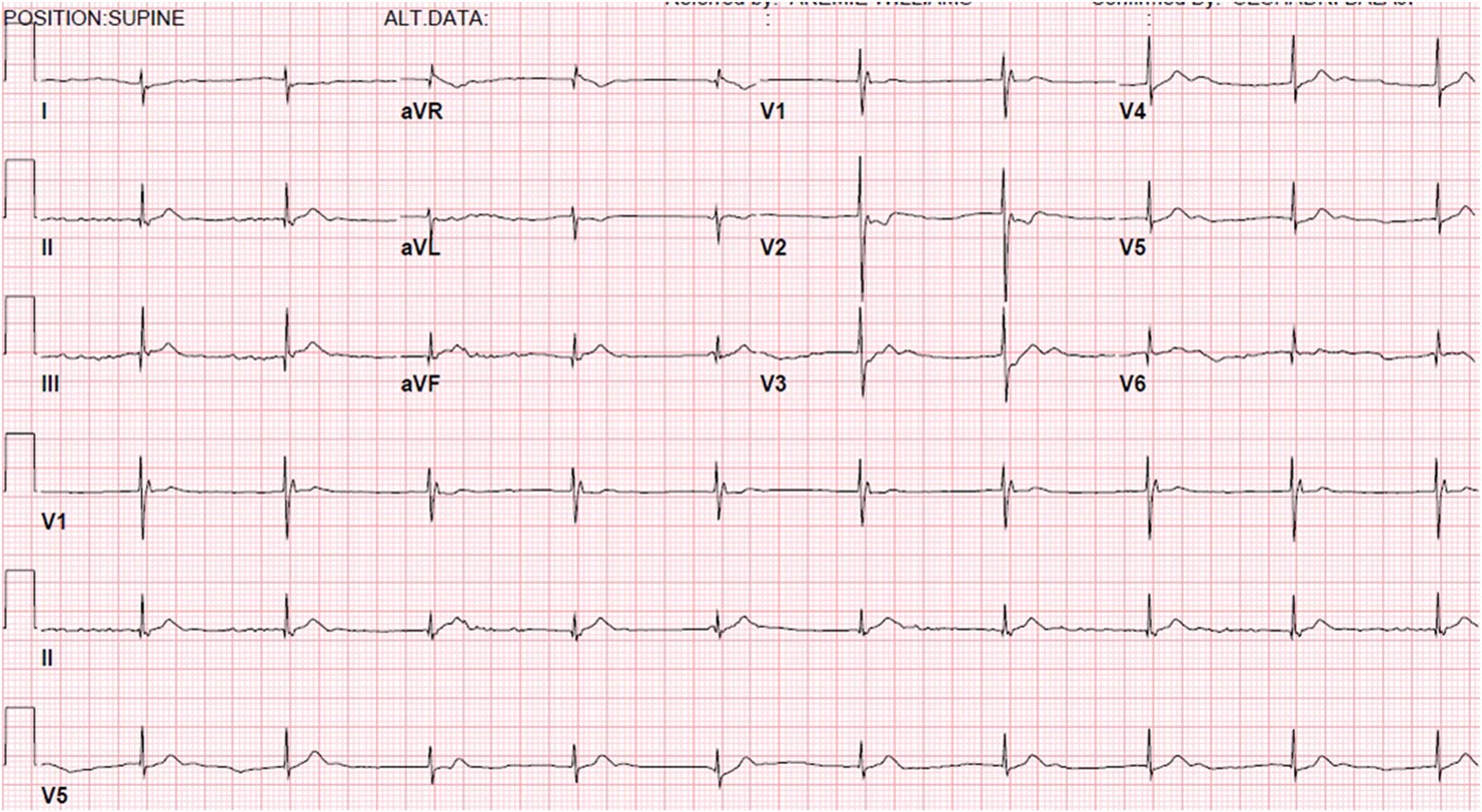
Electrocardiogram on day 1 of life (case 2). Demonstration of junctional bradycardia with a QT/corrected QT interval of 260/314 ms using Bazett correction (heart rate approximately 60 bpm).

At 1 year of age, atrial lead noncapture was noted (Figure 5) and attributed to presumed lead dysfunction, prompting implantation of a dual-chamber epicardial pacing system. AF was not recognized at that time. At 8 years of age, recurrent atrial noncapture prompted invasive evaluation in the catheterization laboratory to help understand his conduction issues. During the procedure, the atrial lead demonstrated noncapture despite high output pacing, and intracardiac electrograms showed him to be in fine AF. Electrical cardioversion was unsuccessful. Because atrial pacing was not possible owing to the persistent AF, a single-chamber endocardial ventricular pacemaker programmed VVIR was implanted.

**Figure 5:**
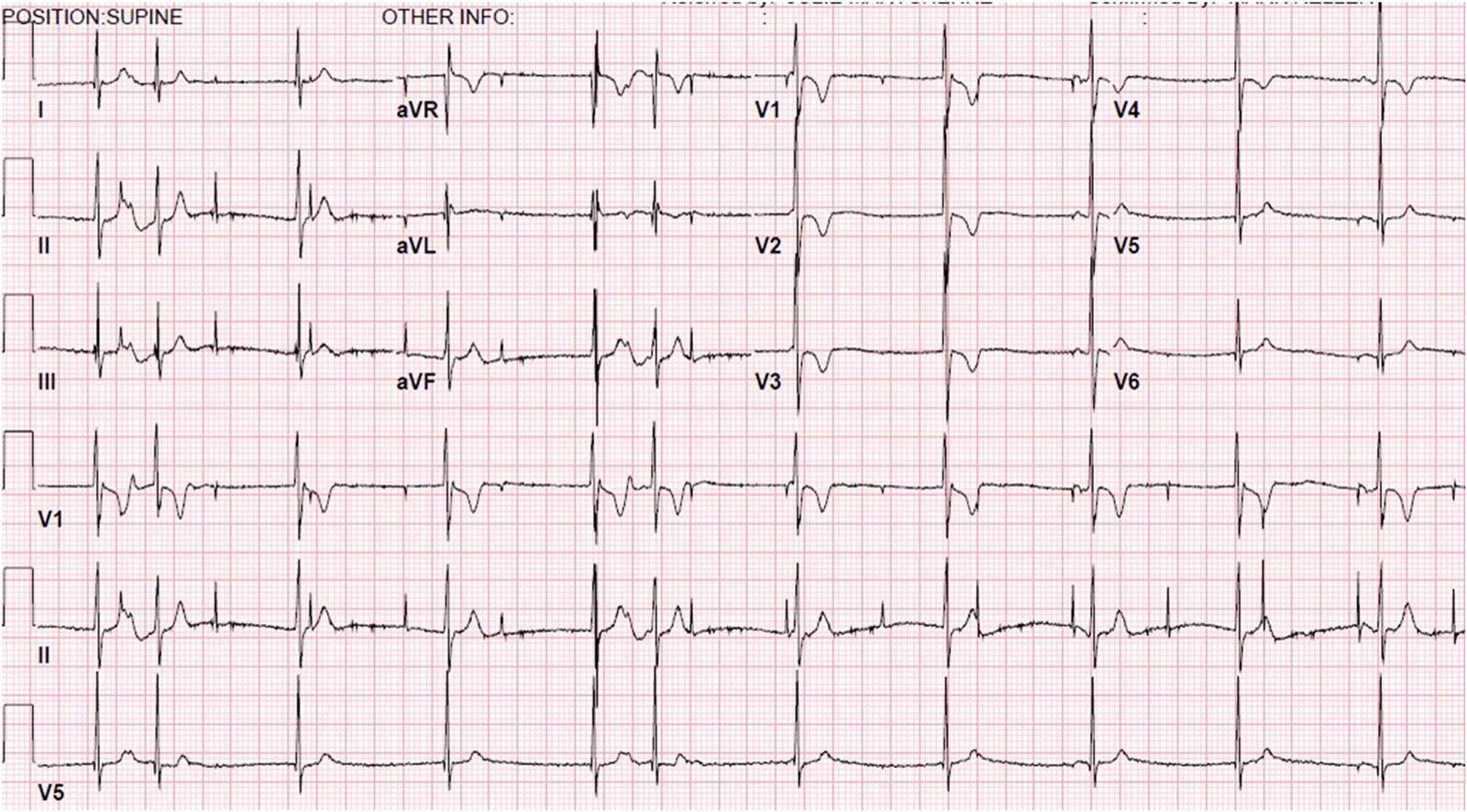
Electrocardiogram at 1 year of age on AAIR pacing (case 2). Atrial lead noncapture and high-frequency atrial signals were demonstrated, which were initially interpreted as lead noise.

Genetic testing identified the same heterozygous *KCNQ1* p.Val141Met mutation seen in case 1. His mother and 2 older siblings were negative for the same gene mutation. His father declined genetic testing. As in the previous patient, implantable cardioverter-defibrillator implantation was discussed but deferred given the absence of ventricular arrhythmias, syncope, or family history of sudden cardiac death, and the patient was discharged on daily aspirin therapy. He remains clinically stable at 17 years of age. A timeline of the pertinent clinical events for both patients is presented in Table 1.

**Table 1:** Timeline for each patient

Event	Case 1	Case 2
Fetal bradycardia	Yes	Yes
Neonatal QTc	307 ms	314 ms
Initial pacing	AAIR	AAIR
Genetic diagnosis	Neonatal period	Neonatal period
AF first detectable	19 mo	1 y
Final pacing mode	VVIR	VVIR
Follow-up	2 y	17 y

AAIR = atrial-pacing atrial-sensing inhibited-response rate-adaptive; AF = atrial fibrillation; QTc = corrected QT interval; VVIR = ventricular-pacing ventricular-sensing inhibited-response rate-adaptive.

## Discussion

These cases illustrate several important clinical features of pediatric SQT2 associated with the *KCNQ1* p.Val141Met variant. Both patients initially presented with congenital sinus node dysfunction as fetal or neonatal bradycardia. Because atrioventricular conduction was preserved and AF was not recognized at the time of initial device implantation, atrial pacing was pursued as an appropriate initial strategy. However, both patients subsequently developed persistent fine AF, rendering atrial pacing ineffective despite otherwise reassuring device parameters.

In retrospect, subtle AF was detectable on earlier ECG recordings in both patients but was initially difficult to distinguish from artifact. In infants, fine AF may generate low-amplitude, high-frequency atrial activity that resembles baseline noise on surface ECG. Failure to recognize this pattern may delay diagnosis and may lead to misinterpretation of apparent pacing system malfunction, particularly atrial lead “noncapture.”

To the best of our knowledge, this represents the first report specifically highlighting recurrent atrial lead noncapture despite stable lead impedance as a manifestation of previously unrecognized fine AF in pediatric SQT2 associated with the *KCNQ1* p.Val141Met variant. The shortened atrial action potential duration resulting from this gain-of-function mutation promotes atrial reentry and predisposes affected individuals to early-onset AF. In retrospect, the apparent atrial lead noncapture observed in both patients likely reflected persistent fibrillatory atrial activity rather than structural lead malfunction. Rapid atrial electrical activity during AF may interfere with effective atrial capture despite stable lead impedance and otherwise normal device parameters.

Recognition of this phenomenon is clinically important. Apparent atrial lead malfunction in children with SQT2 may instead reflect persistent fibrillatory atrial activity. Earlier identification of subtle AF may prevent misinterpretation as hardware failure, avoid lead revisions that may not be beneficial, and facilitate timely consideration of alternative pacing strategies. These cases also highlight important pacing considerations in pediatric SQT2. Although atrial pacing may initially be appropriate in infants presenting with isolated sinus node dysfunction and preserved atrioventricular conduction, progression to persistent AF may ultimately render atrial pacing ineffective. In such circumstances, ventricular pacing strategies may become necessary, as occurred in both of our patients. However, chronic ventricular pacing in pediatric patients carries important long-term considerations, including pacing-induced ventricular cardiomyopathy, repeated lead revisions related to somatic growth, and cumulative device-related morbidity. Accordingly, atrial or dual-chamber pacing strategies may remain reasonable initial approaches in selected infants, with subsequent pacing decisions individualized according to arrhythmia progression, device performance, and evolving clinical status.

Review of the published literature further supports the distinctive phenotype associated with the *KCNQ1* p.Val141Met variant. Among 16 reported cases, affected individuals commonly presented with marked QT shortening (median QTc 292 ms; most ≤330 ms), perinatal bradycardia, sinus node dysfunction, atrial standstill or junctional rhythm, and slow AF. Genetic testing, performed using targeted sequencing with Sanger confirmation, was performed at a median age of 2 years. AF was reported in 87.5% of cases, and among the 7 patients for whom age at AF onset was available, 5 presented before 1 year of age, including 3 diagnosed during fetal life and 2 during infancy. Notably, ventricular arrhythmias were not reported in this cohort.^6^

Management primarily involved pacemaker implantation in 63% of the patients at a median age of 2 years, with ventricular pacing often required owing to atrial standstill. Although optimal pacing strategy remains uncertain, early pacing with a dual-chamber pacemaker may be a reasonable consideration given the distinctive atrial arrhythmia in this population.^6^ Ultimately, device selection should be individualized based on atrial rhythm status, atrioventricular conduction, anticipated disease progression, and the potential long-term consequences of ventricular pacing in young patients.

The structural changes that arise from mutations within cardiac ion channels affect not only their pacemaker activity but also their ability to interact with substances, including medications.^10^ Pharmacologic therapies, including quinidine and hydroquinidine, have been reported in SQTS to prolong repolarization and reduce arrhythmia burden; however, pediatric experience remains limited, with only 4 children (25%) in the SQT2 cohort described earlier receiving quinidine, primarily in conjunction with pacing.^6^^,^^11^ Therefore, further studies evaluating antiarrhythmic responses among individual SQTS variants are warranted. In our patients, antiarrhythmic therapy was considered but not pursued given the predominantly bradyarrhythmic phenotype and absence of ventricular arrhythmias.

Given the rarity of SQT2, evidence guiding optimal pacing strategies in affected children remains limited. These cases expand the recognized phenotype of *KCNQ1* p.Val141Met-associated SQT2 by demonstrating that fine AF may masquerade as atrial lead malfunction despite normal device parameters. Early recognition of this phenomenon may inform pacing decisions, prevent unnecessary lead revisions, and facilitate timely transition to alternative pacing strategies when atrial pacing becomes ineffective.

## Conclusion

We report 2 rare pediatric cases of SQT2 associated with the *KCNQ1* p.Val141Met mutation, presenting with congenital sinus node dysfunction and early-onset AF. These cases highlight the diagnostic challenge of recognizing fine AF in infants, in whom atrial activity may initially resemble artifact on surface ECG.

Persistent AF may compromise atrial pacing efficacy and mimic lead malfunction despite stable device parameters. Awareness of this phenomenon is important when evaluating pacing failure in patients with SQT2. Early recognition of subtle AF and consideration of alternative pacing strategies, including ventricular pacing, may improve long-term management of these complex patients.

## Declaration of generative AI and AI-assisted technologies in the writing process

During the preparation of this work, the authors used Open Evidence to search for published case reports on SQT2 and diagnostic consensus. After using this tool/service, the authors reviewed and edited the content as needed and take full responsibility for the content of the published article.

## Disclosures

Seshadri Balaji is a consultant to Milestone Pharmaceuticals. The other authors have no conflicts of interest to disclose.
